# Patient-tailored reproductive health care

**DOI:** 10.3389/frph.2022.917159

**Published:** 2022-07-18

**Authors:** Jan Tesarik, Raquel Mendoza-Tesarik

**Affiliations:** MARGen Clinic, Molecular Assisted Reproduction and Genetics, Granada, Spain

**Keywords:** fertility, infertility, assisted reproduction, *in vitro* fertilization, personalized strategy, precision medicine

## Abstract

Patient-tailored reproductive health care represents an important challenge for the current practice of infertility prevention, diagnosis and treatment. This approach is based on the concept of precision medicine, taking into account genetic, epigenetic, metabolic and lifestyle characteristics of each individual patient. Even though this goal is still far from being wholly achieved, some aspects can already be put into practice nowadays. Personalization can be based on a comprehensive analysis and synthesis of the patients' personal and familial history, taking into account outcomes of previous assisted reproduction technique (ART) attempts, if available, and confronting these data with the past and the latest clinical and laboratory examination outcomes. As to the male fertility status, there is an urgent need for the inclusion of an accurate diagnostic workup of infertile men leading to the choice of the most adequate follow-up for each particular pathological condition. The follow-up of women who have become pregnant as a result of the ART attempt has also to be personalized. This should be done taking into account both the basic data extracted from the patient's file and those derived from the experience gathered during the latest attempt. Last but not least, the individual condition of each couple has to be taken into account when counseling the patients as to the urgency of the actions to be taken to resolve their fertility problem.

## Introduction

Since the birth of the world's first baby resulting from *in vitro* fertilization (IVF), more than 40 years ago (1), many IVF-derived techniques have been developed and applied in assisted reproduction techniques (ART) to cope with specific issues of male and female infertility (2). However, in spite of these advancements, the overall efficiency of ART still remains relatively low nowadays, and there still persists a lot of room for improvements of ART, especially with regard to cost-effectiveness (3). Thus, efficiency of ART is a problem that needs to be urgently solved, and the way how to achieve this goal will require the recourse to more precise and personalized approaches, tailored to the individual condition of each infertile couple.

According to the definition by the National Institutes of Health (NIH), precision medicine refers to a thorough understanding of the unique features that can influence the existing pathological condition of each individual patient, such as environmental exposures, lifestyle, and genetic profile (3). As to ART, the application of precision medicine means that, instead of treating all patients with the same procedures, this type of personalized health model is expected to increase the efficacy, efficiency and cost-effectiveness of the treatments, and some encouraging outcomes have already been reported in women with low ovarian reserve (4, 5), where the personalized ovarian stimulation protocol improved IVF outcomes, especially in the context of mild ovarian stimulation (6). There are many possible factors that hinder ART outcomes, some of them related to sperm and oocyte quality, and others to the function of different maternal organs whose abnormalities can negatively influence the processes of embryo implantation and subsequent embryonic and fetal development.

This is a short review focusing on data that can serve to increase ART efficiency by personalizing different phases of the process, including the overall planning of the actions to be taken, the preventive measures, the diagnostics, the choice of the ART technique, the adaptation of the ART technique chosen, and the patients' follow-up after embryo transfer,

## Patient-tailored reproductive health care: A call for action

Patient-tailored medicine is the first, but important, step toward the use of precision medicine. Precision medicine can be considered as a systematic application of molecular medicine, by creating a patient specific imprint of genetic, epigenetic, transcriptomic and metabolomic traits for each patient, and the use of these data in the choice of methods to be used (3). This need is based on the current evidence of differences between individual men and women as to their response to different types and doses of medicines used for the solution of their fertility problem (4–6). The idea means that each case of infertility should be managed in a personalized way instead of applying the same regimens to everybody. However, we are still far from a strict application of personalized medicine, based on the knowledge of the complete and precise molecular profile of each patient. In this paper we resume the current possibilities of orienting our actions in this sense as well as the main challenges for future molecular and clinical research to go ahead in this direction.

## Personalized medicine–the definition

Personalized (or precision) medicine is based on the idea that different individuals have unique and particular characteristics at the molecular, metabolic and environmental exposure levels. Consequently these particularities should be taken into account to provide diagnostics and therapeutic interventions that are tailored to these nuances and unique personal characteristics (7). Despite initial doubts about the possibilities of applying precision medicine in human reproductive care (7), new data show the way how to achieve this goal at medium and long term (8, 9). Anyway, even before having a complete guide, things can be done in this sense by using clinical and biological data already available nowadays.

## What goals can be achieved nowadays

Even in the absence of a complete molecular record, some aspects of personalized medicine can already be incorporated into the infertility management strategies nowadays. These aspects should be based on a complete medical record, including male physical examination and female pelvic ultrasound with hormonal evaluation as the primary basis of investigation. In addition, the age of both partners, their personal and familial history, as well as the presence of eventual comorbidities are also to be taken into account (10).

## Personalized approach to infertility management

The personalized approach to infertility management should comprise different successive stages of the decision-making processes, including personalized strategy, personalized preventive measures, personalized diagnostics, and personalized treatments, as well as post-treatment follow-up.

### Personalized strategy

**Figure 1:**
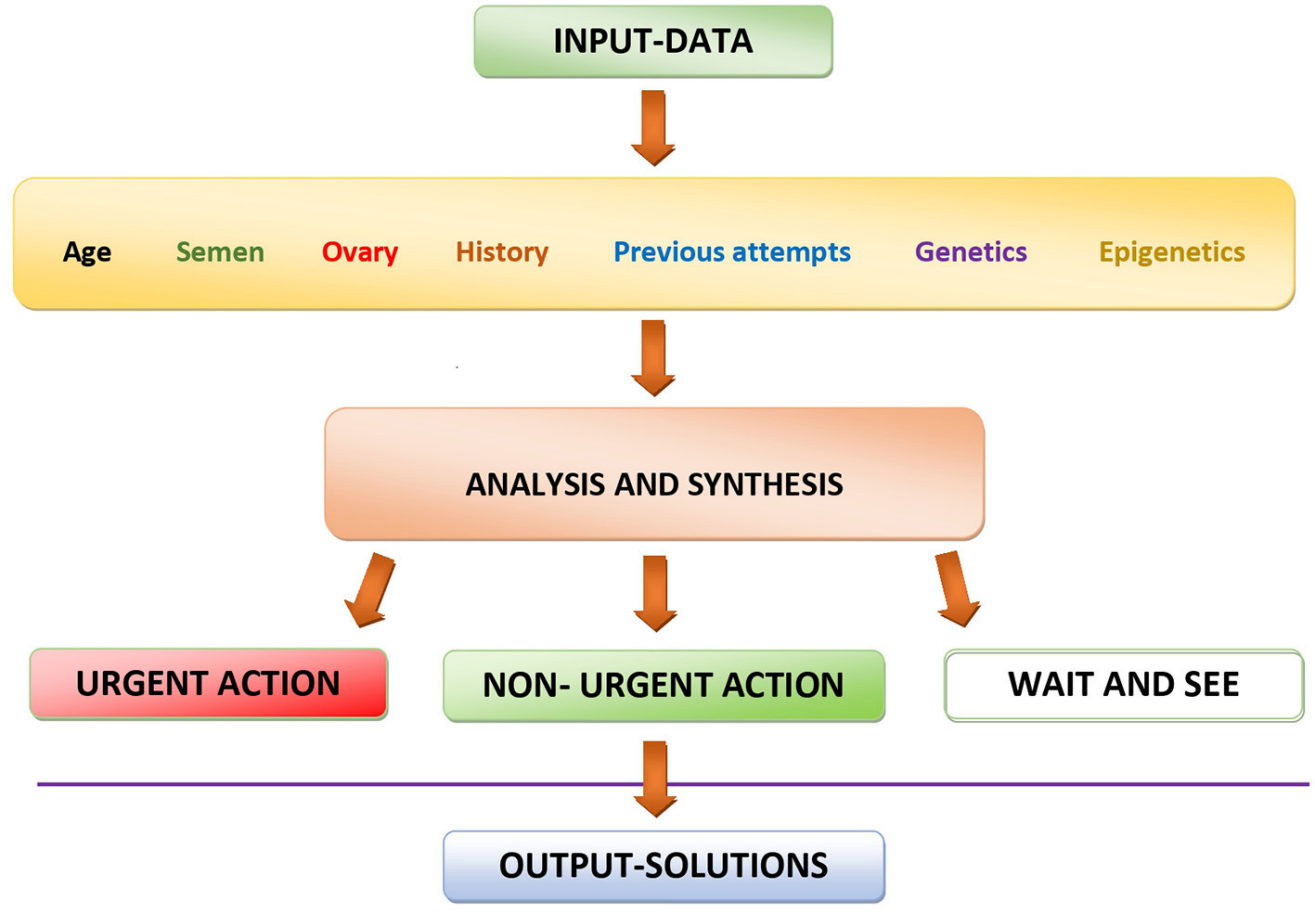
Schematic representation of the decision-making process using currently available data for personalizing diagnostic and therapeutic actions in the management of infertility.

The optimal strategy will obviously depend on the specific condition of both partners of an infertile couple (Figure 1). For younger persons with relatively short duration of infertility, a “wait-and-see” approach may be the first option, in order to avoid unnecessary cost related to the use of diagnostic methods. However, if the infertility persists, something has to be done, especially in women with advancing age and in men whose personal and familial history and/or whose life-style factors can be suspected to cause spermatic alterations.

The initial evaluation of the male partner should include medical history, physical examination, and semen analysis. Semen microbiological examination, endocrine assessment and testicular ultrasound imaging should be suggested in all cases. They should be strongly recommended when specific risk factors for infertility exist or first-step analyses showed abnormalities. Genetic tests, testicular cytology/histology and additional tests on sperm, such as the evaluation of sperm DNA integrity, are clinically oriented and based on the results of previous investigations (11).

For women, the diagnosis should begin with vaginal ultrasound examination and basic hormonal analyses. These examinations may be complemented by hysteroscopy in cases in which the patient has a history of endometrial polyps or of previous intracavitary interventions, especially endometrial scratching, curettage or induced abortion.

### Personalized preventive measures

If the basic examinations of one or both partners show abnormal results, personalized preventive measures can be envisaged. Their choice will depend on the analysis of all potential harmful factors that could impair the ovarian and testicular function. To begin with, all known factors leading to reproductive impairment should be searched for. There is an ample spectrum of such factors, including genetic predisposition, epigenetic abnormalities, life-style factors (such as smoking or alcohol and drug abuse), professional exposures to gametotoxic substances, the use of anabolics and other hormones to boost the performance in persons involved in high-level professional sport activities, and a number of comorbidities, such as abnormal function of the thyroid gland or insulin resistance. Most of this kind of abnormalities can be resolved relatively easily by appropriate medications and life-style changes.

On the other hand, if no underlying cause can be detected, the situation becomes more complicated. Both the testicular (12, 13) and ovarian (10) functions can be impaired by factors of epigenetic origin that are less easily detectable than genetic defects. It was shown that many of such undetected etiological factors converge to oxidative stress, both in spermatozoa and oocytes, as well as in associated testicular and ovarian cells whose dysfunction can affect the gamete quality indirectly (13, 14). This is the reason why oral treatment with antioxidants is being increasingly used in both the prevention and treatment of such conditions (14). However, it is important to realize that a shift of the redox balance from the oxidative stress toward the opposite extreme, the reductive stress, can be equally counterproductive with regard to the pathology treated. Moreover, reductive stress, caused by abusive intake of antioxidants, can be dangerous for other, unrelated functions of the organism (14).

### Personalized diagnostics

Personalized diagnostics are basically part and extension of the methods used for the choice of preventive measures, as mentioned in the previous section. Genetic and epigenetic factors involved in early embryo demise have been extensively reviewed recently (15, 16). However, none of them appears to be a unique culprit, so that a combination of different factors, rather than just one, appears to be responsible. There is a general agreement that no embryo should be discarded on the basis of the detection of one or a few abnormalities because embryos can still maintain developmental competence, due to factors that are still not fully understood and appear to be highly personalized, too (15, 16).

### Personalized treatments

**Figure 2:**
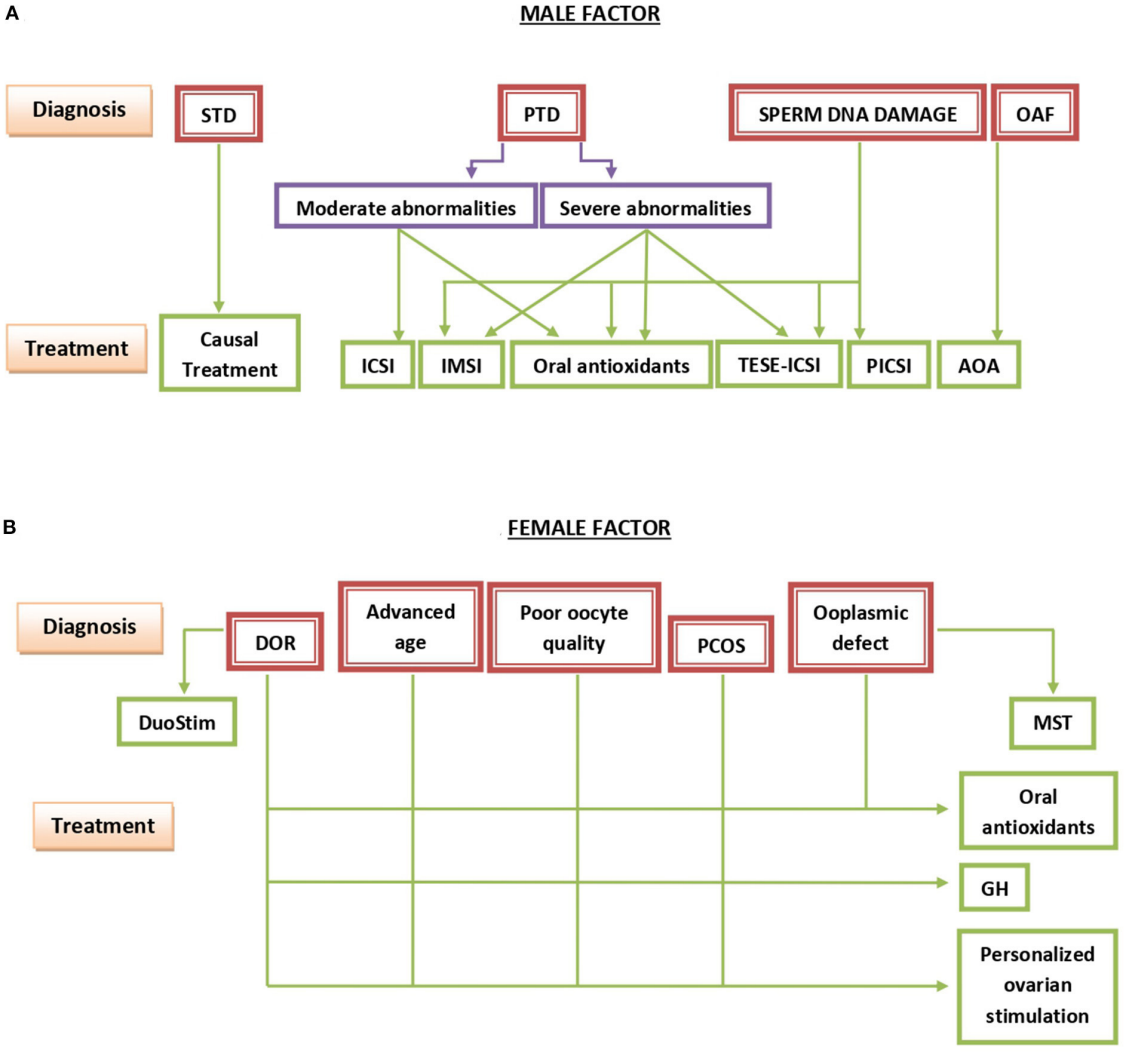
Summary of the basic points outlining the management of infertility due to the male **(A)** or the female **(B)** factor. STD, secondary testicular disease; PTD, primary testicular disease; OAF, oocyte activation failure; ICSI, intracytoplasmic sperm injection; IMSI, intracytoplasmic morphologically-selected sperm injection; TESE-ICSI, ICSI with spermatozoa recovered by testicular sperm extraction; PICSI, physiologic ICSI; AOA, assisted oocyte activation; DOR, diminished ovarian reserve; PCOS, polycystic ovary syndrome; DuoStim, double ovarian stimulation; MST, meiotic spindle transfer; GH, growth hormone.

Personalized treatment strategy involves two important decisions. First, the emergency of the treatment and, secondly, the type of treatment chosen. Both of these decisions should be based on a thorough evaluation of the particular condition of each individual couple (Figure 1) and can go from a simple wait-and-see approach through relatively “light” medications up to much more complicated and costly assisted reproduction techniques (Figure 2).

#### Choice of treatment method

In cases of young couples, with relatively short infertility duration, the wait-and-see approach can be the best solution. If there is no spontaneous improvement, different medications, combined with appropriate clinical and laboratory controls, can be chosen to better synchronize the days of sexual intercourse with the embryo implantation window (the optimal time for the maternal organism to promote embryo implantation in the uterus). If this approach does not give satisfactory results, it will be necessary to have a recourse to more sophisticated methods, going from artificial insemination through IVF up to intracytoplasmic sperm injection (ICSI), always taking into account that the more sophisticated the method of choice is, the more costly it will be for the couple (if performed in a private clinic) or for the social security (if performed an a state-owned clinic). Hence, maximal attention is to be paid to the eventual adaptations of the treatment method chosen, taking into account the overall condition of the couple concerned (Figure 1).

#### Adaptation of the treatment method chosen

For men with slight or moderate sperm abnormalities, conventional ICSI is the method of choice, while severe teratozoospermia or a high degree of sperm DNA fragmentation, the recourse to intracytoplasmic morphologically selected sperm injection (IMSI) is preferable (17). For men with different degrees of sperm DNA damage, an algorithm of *in vivo* and *in vitro* treatments, going from a simple oral treatment with antioxidants through ICSI, physiologic ICSI (PICSI) selecting spermatozoa based on their capacity to bind hyaluronic acid, IMSI, up to ICSI with spermatozoa recovered by testicular biopsy—testicular sperm extraction followed by ICSI (TESE-ICSI), according to the severity of the condition, has been suggested (13). TESE-ICSI is also indicated in men with extreme oligoasthenoteratozoospermia or cryptozoospermia. In men in whom semen abnormalities are due to a known cause, such as hormonal imbalance or varicocele, this should be corrected first before having recourse to more complicated and costly procedures (Figure 2).

For women close to, or over, 40 years of age, the use of growth hormone during ovarian stimulation is of advice, in order to improve the quantity and quality of oocytes recovered by ovarian stimulation (18).This same treatment is recommended for women with poorly developing endometrium (19) and for those in whom suboptimal oocyte and endometrial quality is due to polycystic ovarian disease (20). Other adaptations can be taken as a result of a thorough analysis of all factors potentially involved in the existing infertility case, originating from both the male and the female condition (Figure 2).

The use of antioxidants to protect energetic metabolism of oocytes and somatics cells of the ovary is also recommended. The choice of the type of the antioxidant drug, the duration of the treatment and the dose are all to be determined in a personalized way, always taking into account that excess use of antioxidants can be counterproductive and even dangerous for the general health status of the patient treated (14). The optimal decision requires a thorough analysis of different aspects of each case (14), including the age, ovarian reserve, the personal and individual history of the couple, the results of eventual previous treatment attempts and, if available, the data of the genetic and epigenetic status of the patient (Figure 2).

Advanced female age being increasingly important as a cause of human infertility nowadays, there are other, more sophisticated clues with which this problem can potentially be addressed. Actually, even though both physiological and premature ovarian aging usually result in oxidative stress caused by mitochondrial damage (21), there are many developmentally important molecules in the oocyte cytoplasm that are much more exposed to oxidative stress than the oocyte's DNA which is relatively protected by its intranuclear location and association with proteins.

More than two decades ago, nuclear transfer from aging women into enucleated donor oocytes was shown to be able to save fertility without sacrificing the women's own genome, as would be the case of complete oocyte donation (22). As an alternative, attempts were made at rejuvenating oocytes by intracytoplasmic injection of a small amount of donor oocyte cytoplasm into the patients's oocytes at the time of IVF carried out by intracytoplasmic sperm injection (ICSI) (23). Both methods were shown to improve embryo development and embryo outcomes in patients with previous problems, including those of advanced age (24, 25). With the use of each of them, several tens of healthy babies were born. However, because of still poorly defined and unexplainable reasons, this type of oocyte cytoplasmic manipulation was banned, first in the US and then in most European countries. Only since a couple of years ago, the original method of nuclear transfer (22) began to be used again in countries in which no prohibition existed. However, its reuse was initially limited to relatively young women carrying previously detected mutations of oocyte mitochondrial DNA, thus avoiding the initial question of whether this technique can be of help for women of advanced age (22–25). This question has to be re-addressed, namely in view of experimental studies suggesting that nuclear transfer can rejuvenate aged animal oocytes (26–28).

#### Ovarian stimulation protocol

The choice of the optimal ovarian stimulation protocol is another issue to be addressed. We know that there are interindividual differences as to the response of women to different types of hormones and their combinations (29, 30), and the first studies into the molecular basis of these differences begin to emerge (31). However, for the time being, here again clear guides and protocols are still missing, and the ovarian stimulation protocol should thus be mainly adapted according to the results of previous attempts, in addition to repeated determinations of serum estradiol and LH concentrations, as described previously (32). Though initially discovered by studying oocyte quality in young oocyte donors, this issue is even more important in women with diminished ovarian reserve, as described more recently (5). Evidently, there is an urgent need for performing more high-quality molecular and clinical studies to address this point so as to be able to apply the optimal ovarian stimulation protocol as early as the first treatment attempt.

Last but not least, double ovarian stimulation (DuoStim) in the follicular and luteal phase of the same cycle, followed by oocyte or embryo cryopreservation for later uterine transfer, is increasingly used in women with extremely poor ovarian response (POR) and asynchronous antral follicle growth during the follicular phase (29). A recent systematic review and meta-analysis has suggested that this option may present as a promising method in the management of POR patients by enabling a higher oocyte yield during a single menstrual cycle (33).

Figure 2 shows a schematic representation of diagnostic and therapeutic choices to be taken in cases of infertility caused primarily by the male (Figure 2A) or the female (Figure 2B) factor. Briefly, when a secondary testicular disease (STD) is detected, the causal treatment of the primary cause is indicated, On the other hand, the decision-making scheme in cases of the primary testicular disease (PTD) is a much more complicated issue with a number of alternative treatments to be taken into consideration. If spermatozoa only show moderate abnormalities, conventional ICSI is sufficient in most cases. On the other hand, in cases of severe sperm abnormalities, the recourse to IMSI or even TESE-ICSI is preferable. TESE-ICSI is especially necessary in the most severe cases, such as cryptozoospermia. In cases of abnormally elevated concentrations of spermatozoa with damaged DNA, IMSI, PICSI, or both in combination can be of help. However, in the most severe cases TESE-ICSI is recommended. In all above mentioned conditions, oral treatment with antioxidants for at least 2 months usually leads to an additional improvement of sperm DNA integrity. Finally, in cases in which problems of oocyte activation after sperm deposition in oocyte cytoplasm has previously been observed, different methods of assisted oocyte activation (AOA) usually resolve the problem (Figure 2A).

As to the female factor, therapeutic solutions of diminished ovarian reserve (DOR), issues related to advanced maternal age, poor oocyte quality at any age, and polycystic ovarian syndrome (PCOS) converge to the use of growth hormone (GH) during ovarian stimulation and the choice of a personalized protocol of ovarian stimulation adapting the doses of FSH and LH to be administered according to the current FSH-to-LH ratio in serum at different sequential phases of the stimulation. Oral antioxidants are of help in all of the above conditions. Moreover, double ovarian stimulation (DuoStim) and meiotic spindle transfer (MST) are useful in patients with DOR and with different ooplasmic defects, respectively (Figure 2B).

Last but not least, despite much effort spent to date to identify a correct algorithm which considers woman's age and ovarian reserve markers as a tool to optimize the recombinant follicle-stimulating hormone (rFSH) starting dose in IVF procedure, current available evidence regarding PCOS women, particularly the ones with high AMH, does not seem adequate and further research is needed to optimize the ovarian stimulation protocol in this category of patients (34). For instance, it has been shown that obesity, a condition often associated with PCOS, is associated with significantly higher expression of progesterone receptor and pentraxin 3 in the cumulus cells, and obese women require twice as much additional gonadotropins for ovarian stimulation to achieve similar IVF success rates as normal-weight women (35).

#### Post-treatment follow-up

The follow-up of women after the ART attempt is another issue which is still underestimated nowadays. Contrary to earlier opinions suggesting that luteal phase deficiency is a relatively marginal issue, more recent data suggest that this is not the case, even in natural conception cycles (36), and the problem is even more important after ART attempts using different ovarian stimulation protocols (37, 38). Moreover, in addition to the luteal phase itself, the luteoplacental shift (the relief of the role of the corpus luteum by the placenta as the main source of progesterone) also appears to be disturbed in some women after ART treatments (39), and prolonged administration of progesterone during pregnancy may be useful when serum progesterone levels after the supposedly completed luteoplacental shift begin to fall (40).

A long-term follow-up of male patients with different pathological conditions (varicocele, Klinefelter syndrome or other primary testicular disease) is also important. If not corrected before treatment, surgical treatment of varicocele is recommended to prevent further impairment of sperm quality and quantity. If no specific treatment of the existing primary testicular disease is available, fertility preservation by freezing a sample of ejaculated spermatozoa or of surgically retrieved testicular tissue should be considered in order to facilitate eventual future ART attempts. Secondary testicular failures due to endocrine imbalances should be treated by appropriate treatment of the primary cause because, if untreated, hormonal imbalance may also cause long-term disturbances in different tissues and organs other than the testis.

## Patient categorization and determination of priorities

In any case, careful analysis and synthesis of biological and clinical data of each infertile couple always was of utmost importance (Figure 1), and this aspect is even more important in the current COVID and post-COVID era, when many treatment cycles have had to be canceled or delayed due to COVID-related restrictions (41). All aspects of each case, both of the male and the female origin, have to be taken into account to determine the priorities of treatment in each individual case, determining which couples need an urgent treatment from those whose treatment is less urgent and those in whom the wait-and-see approach is the best choice (Figure 1).

## Limitations and strengths

The limitation of this paper is that the coverage of the issues related to the topic is incomplete because the paper is intended to encourage more authors to publish, including both original research data and more complete reviews. The strength can be resumed as an invitation for using personalized data of each infertile couple to tailor the protocol of their infertility management, instead of having recourse to standard protocols.

## Conclusions

The current success rates of human assisted reproduction are still rather low as compared with other species. This can be partly due to the inherently lower reproductive performance of the human species as compared to animals. The increasing female age is another factor which comes into play. However, these two aspects should not be blamed as the only culprits. Evidence is accumulating to show that each infertile couple has specific needs which have to be taken into account in choosing the optimal diagnostics, the optimal clinical and laboratory procedures, and the optimal post-treatment follow-up. The more complicated is the case, the more important is the personalized approach. While more high-quality molecular and clinical studies still have to be carried out to introduce precision medicine principles into human assisted reproduction, we already can use information from the patients' history, previous attempts and available genetic and epigenetic data to adapt the treatment to each couple's condition in the optimal way possible.

## Author contributions

JT and RM-T were contributed equally to the conception, writing, and editing of the article. All authors contributed to the article and approved the submitted version.

## Conflict of interest

The authors declare that the research was conducted in the absence of any commercial or financial relationships that could be construed as a potential conflict of interest.

## Publisher's note

All claims expressed in this article are solely those of the authors and do not necessarily represent those of their affiliated organizations, or those of the publisher, the editors and the reviewers. Any product that may be evaluated in this article, or claim that may be made by its manufacturer, is not guaranteed or endorsed by the publisher.
